# Efficacy and Safety of Centchroman in the Treatment of Breast Fibroadenoma: A Systematic Review and Meta-Analysis

**DOI:** 10.7759/cureus.87046

**Published:** 2025-06-30

**Authors:** Keshav Kumar, Kumari Pallavi, Kumar Martand, Lalit Mohan, Sukalyan Saha Roy, Prem Prakash, Vikas Maharshi

**Affiliations:** 1 Department of Pharmacology and Therapeutics, Indira Gandhi Institute of Medical Sciences, Patna, IND; 2 Department of Anatomy, Employees State Insurance-Post Graduate Institute of Medical Sciences and Research (ESI-PGIMSR) Employees State Insurance Corporation (ESIC) Medical College, Joka, Kolkata, IND; 3 Department of Surgery, Indira Gandhi Institute of Medical Sciences, Patna, IND; 4 Department of Pharmacology, All India Institute of Medical Sciences, Patna, IND

**Keywords:** breast fibroadenoma, centchroman, meta-analysis, ormeloxifene, systematic review

## Abstract

Breast fibroadenoma is a benign disease in women of reproductive age group which usually presents as painless, solitary, or multiple lumps located in the same or both breasts. Management of breast fibroadenoma ranges from watchful observation for self-regression to surgical interventions. Many studies have evaluated centchroman for its efficacy and safety in the treatment of breast fibroadenoma, but their findings were conflicting. We, therefore, aimed to conduct this systematic review and meta-analysis to resolve the conflict. The primary objective was to assess the efficacy whereas the secondary objective was to assess the safety of centchroman compared with no intervention-only watchful waiting, placebo or any active control in patients with breast fibroadenoma.

The protocol for this systematic review and meta-analysis was designed *a priori *and registered in PROSPERO (CRD42024552160). Randomized controlled trials (RCTs), non-randomized interventional studies and prospective observational studies that evaluated efficacy and safety of centchroman in patients of breast fibroadenoma were included in the systematic review whereas only RCTs were included in the meta-analysis. Review articles, case reports and communications were excluded. A comprehensive search of databases such as Medline, Science Direct, Cochrane Library and Google Scholar and trial registries such as ClinicalTrial.gov and Clinical Trials Registry-India (CTRI) till 31/12/2024 was carried out by two independent authors. The efficacy outcome was regression of fibroadenoma measured in terms of the number of responders in each arm whereas the safety outcome was the number of patients who developed any adverse reaction. Risk ratio (RR) was used as effect measure and random effect model was used for data analysis.

During the preliminary search, we found 8113 search results, of which 60 remained after removing irrelevant and duplicate results and the results available in non-English languages. We obtained full texts of 26 studies that were screened further for eligibility. Eventually, seven studies were eligible for systematic review, of which two studies were included in the meta-analysis. In the meta-analysis of two RCTs with pooled sample size of 234, the pooled estimate (RR) of efficacy outcome was 1.44 [95% CI 0.59 to 3.50; *p*=0.42] and heterogeneity was found to be moderate with *I*^2^ value equal to 46%. The pooled estimate (RR) of safety outcome was 13.08 [95% CI 3.72 to 46.02; *p*=0.0001].

With these findings of meta-analysis, we conclude that centchroman is as efficacious as the control in causing regression of fibroadenoma and centchroman has significantly higher number of adverse effects than the control, although none of the adverse effects were serious. More RCTs with larger sample size and from diverse geographical settings are needed to generate more robust evidence and to expand generalizability over a broader population.

## Introduction and background

A breast fibroadenoma is a painless, benign tumor that presents as a solid lump. It is usually solitary but can be multiple and located in either the same or both breasts [1,2]. Fibroadenoma most commonly occurs in women of reproductive age between 15 and 35 years but may occur at any age, with a reported incidence of 27.6% in women aged 18-40 years [2,3].

The exact cause is yet to be determined, but reproductive hormones such as estrogen and progesterone, family history, race and ethnicity, and genetic factors are well-recognized risk factors [2-8]. Fibroadenoma is a benign lesion with a meager risk of malignant transformation; however, women with a first-degree relative with breast cancer are at increased risk and should be carefully monitored [9,10].

The most common clinical presentation is a palpable mass, which is usually painless, but some women may complain of breast discomfort or pain. The size of the lump varies from a few millimeters to many centimeters. A fibroadenoma is a well-circumscribed, unencapsulated lesion that does not infiltrate adjacent parenchyma. It has a glandular (epithelial) and a stromal (connective tissue) component. Immunohistochemistry reveals the expression of estrogen (ER) and progesterone (PR) receptors on these tissues [11,12].

The diagnosis of fibroadenoma relies on triple assessment, which encompasses clinical examination, imaging, and biopsy. In most cases clinical examination and imaging are sufficient. Biopsy is added when findings from breast examination and imaging are inconclusive or when the lesion looks suspicious of malignant transformation. Clinical breast examination involves palpating the breast mass for its location, consistency, and mobility, which aid in definitive diagnosis. The selection of suitable imaging techniques depends upon the woman’s age and is mostly based on either mammography or breast ultrasound. Fine needle aspiration (FNA), being less invasive and less scarring, is the first biopsy approach but is limited by the small sample volume. In cases where the results of FNA are inconclusive, core needle biopsy is performed. Fibroadenomas have a variable natural history; some regress spontaneously, some remain stable, and others increase in size. The factors that guide the treatment-seeking behaviors of women are symptoms, cosmetic concerns, and fear of malignant transformation [13-15].

Treatment is based on shared decision making and should be performed on a case-to-case basis. There is no approved medical therapy available to date. Since fibroadenoma carries very little risk of malignant transformation, watchful waiting for spontaneous regression is the most common approach for most cases, particularly in smaller asymptomatic fibroadenomas. Surgical interventions such as excisional biopsy or lumpectomy are preferred when the lump size increases or when there is high clinical or radiological suspicion or when a woman is willing for it to be removed for her peace of mind. Cryoablation, ultrasound-guided vacuum-assisted biopsy and radiofrequency ablation are minimally invasive and the least scarring surgical procedures [16-19].

Centchroman (also known as ormeloxifene) is a nonsteroidal, selective estrogen receptor modulator (SERM), developed by the Central Drug Research Institute, Lucknow, India, and was approved by the Central Drug Standard Control Organization (CDSCO), India, in 1990 as an oral contraceptive. It has agonistic action on the endometrium and strong antagonistic action on the breast duct-lobular epithelium. Menstrual irregularities such as the absence of menstruation, scanty menstruation, and delayed menstruation are the most reported side effects [20,21].

The potential of centchroman for the treatment of fibroadenoma has been evaluated in many randomized controlled trials (RCTs), nonrandomized trials and observational studies, but their findings are conflicting. This systematic review aims to present a list of those studies with important findings and to resolve the conflict by performing a meta-analysis.

Primary objective

The primary objective was to assess the efficacy of centchroman compared with no intervention - only watchful waiting, placebo or any active control in patients with breast fibroadenoma.

Secondary objective

The secondary objective was to assess the safety of centchroman compared with no intervention - only watchful waiting, placebo or any active control in patients with breast fibroadenoma.

## Review

Methodology

Registration

The protocol for this systematic review and meta-analysis was designed a priori and registered in PROSPERO (CRD42024552160). This systematic review and meta-analysis were conducted in accordance with the guidelines of the Cochrane handbook for systematic reviews of interventions.

Eligibility Criteria

In this systematic review and meta-analysis, we included only original research articles. Randomized controlled trials, nonrandomized interventional studies and prospective observational studies that evaluated the efficacy with or without the safety of centchroman in patients with breast fibroadenoma were included. Review articles, case reports and communications were excluded. Studies involving women of all ages who received centchroman in any formulation, dose, frequency, or duration as an intervention/test drug and no intervention - only watchful waiting, any active drug or placebo as a comparator/control for breast fibroadenoma, were included in the meta-analysis. 

Outcome Measures

The efficacy outcome was regression of fibroadenoma measured as the number of responders in the centchroman and control groups whereas the safety outcome measure was the number of patients who developed any adverse reaction.

Search Strategy and Selection of Studies

Databases such as PubMed, Google Scholar, Science Direct and the Cochrane Library and trial registries such as ClinicalTrials.gov and the Clinical Trials Registry-India (CTRI) were searched from their inception until 31/12/2024 (see Appendix). Free-text and MeSH terms were used to apply keywords. The search strategy was restricted to the English language only. Depending upon the databases/registers searched, we used the following keywords individually or in combination with suitable Boolean operators: ‘centchroman’, ‘ormeloxifene’, ‘fibroadenoma’, and ‘benign breast disease’.

Two authors (KK and KP) independently carried out the literature search and reviewed the titles and abstracts of the identified articles to identify relevant articles. Duplicates were removed, and articles meeting selection criteria were included for further review. A full-text review of these articles was subsequently performed. Any disputes pertaining to selection were resolved by consultation and mutual discussion with a third reviewer (VM). 

Risk of Bias Assessment

Two reviewers (KK and KM) independently assessed the risk of bias. Assessment of the risk of bias in RCTs was performed via the Cochrane Risk of Bias 2 (RoB 2) tool version beta 9.

Publication Bias

Publication bias in the included studies was checked via a funnel plot.

Data Acquisition

The data collection form was designed in accordance with guidelines of the Cochrane handbook of systematic review for interventions. The data was extracted from individual studies and entered into an Excel (Microsoft, Redmond, WA, USA) sheet by two reviewers (KK and SSR) independently and any discrepancy in acquired data was resolved by discussion with a third reviewer (VM). The collected data consisted of: (a) study-related information (name of author(s), registration, study design, study site, study duration, duration of follow-up, level of blinding, method of randomization and allocation concealment if applicable); (b) participant-related information (total number, number in intervention/exposure and control arms); (c) intervention/exposure-related information (name, dose, frequency and duration of drugs in intervention/exposure and control arms); and (d) outcome-related information (primary and secondary outcomes, number of responders and number of adverse effects in each arm).

Data Analysis

Meta-analysis was performed using Review Manager version 5.4.1 (RevMan 5.4.1). Risk ratio (RR) with 95% confidence interval (CI) was taken as effect measure and the analysis was performed using the random effect (RE) model (DerSimonian and Laird method) as it considers variability among studies. A *p*-value <0.05 was considered statistically significant. Heterogeneity was assessed by the Chi square (Q statistic) and *I*^2^ test and interpreted as low, moderate, substantial, and considerable for *I*^2^ values <25%, 25% to <60%, 60% to <75% and ≥ 75% respectively.

Results

**Figure 1:**
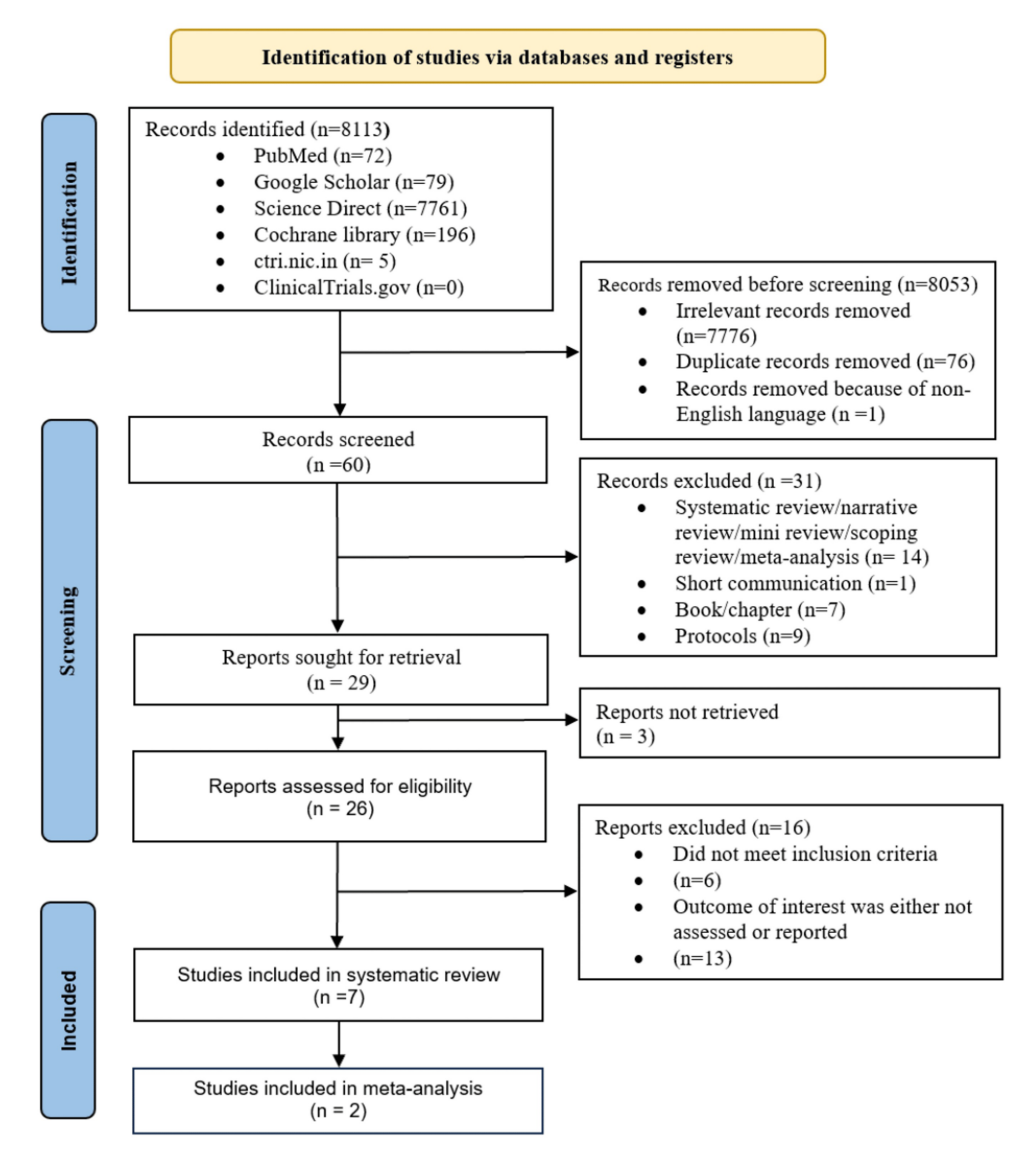
Preferred Reporting Items for Systematic reviews and Meta-analyses (PRISMA) flow diagram showing the selection of studies

We reported results of this systematic review and meta-analysis following the Preferred Reporting Items for Systematic reviews and Meta-analyses (PRISMA) 2020 guidelines (Figure 1). During the preliminary search, we found 8113 search results of which 60 remained after removing irrelevant and duplicate articles and articles in non-English languages. We got the full text of 26 studies which were screened further for eligibility. Eventually, seven studies [22-28] were eligible for systematic review, of which two studies were included in the meta-analysis. Important characteristics of studies included in the systematic review and meta-analysis are presented in Table 1.

**Table 1:** Important characteristics of studies included in systematic review and meta-analysis ^a^Studies included in meta-analysis

Study identifier	Study design & site	Study duration	Inclusion criteria	Sample	Intervention with dosage	Control with dosage	Duration of follow-up	Primary outcome measure (Efficacy)	Secondary outcome measure (adverse effects)
^a^Agrawal et al. 2024 (CTRI/2023/03/050286) [The FIBROCENT study] [22]	Double-blinded, randomized, placebo-controlled trial; India	From March 2023 to October 2023	Patients with biopsy proven simple fibroadenoma with lump size < 5 cm	Total no. of patients with fibroadenoma (N=130) Ormeloxifene group n=65 Placebo group n=65	Tab. Ormeloxifene (30 mg) alternate day for 3 months	Placebo	6 months	Number of patients which showed complete regression Ormeloxifene group n=6 Placebo group n=7	Menstrual irregularity Ormeloxifene arm n=10 Control arm n=2 Dizziness Ormeloxifene arm n=8 Control arm n=0 Ovarian cyst Ormeloxifene arm n=2 Control arm n=0 Heavy bleeding after stopping the drug Ormeloxifene arm n=1 Control arm n=0
^a^Rai et al. 2024 (CTRI/2023/07/055015) [23]	Parallel-arm randomized controlled trial; India	1 year	Patients aged between 18 and 45 years and having single or multiple fibroadenomas ≤ 3 cm	Total no. of patients with fibroadenoma (N=104) Centchroman group n=52 Placebo group n=52	30 mg centchroman on alternate days for 3 months	Placebo (Tab. Calcium 250 mg) on alternate days for 3 months	12 weeks	Number of patients which showed reduction of fibroadenoma volume >50% Centchroman group n=15 Control group n=7	Scanty menstruation Centchroman group n=9 Placebo group n= 0 Delayed menstruation Centchroman group n= 6 Placebo group n= 0
Tejwani et al. 2015 [24]	Open-label, two-arm, parallel design, randomized controlled trial; India	From November 2004 to November 2007	Diagnosed cases of fibroadenoma aged ≤ 30 years	Total no. of fibroadenomas (N=121) Centchroman arm n=69 Control arm n=52	Centchroman 30 mg daily for 12 weeks	Natural observation	24 weeks	No. of fibroadenomas which showed complete regression Centchroman arm n=22 Control arm n=4	Menstrual abnormalities such as missed menstruation and prolongation of menstrual cycle Centchroman arm n=9 Control arm n=0
Sinha et al. 2024[25]	Prospective, Hospital based-observational study; India	1 year	Women aged 16 to 35 years having fibroadenoma of size ≤4 cm	100 women experiencing breast pain, with or without lump, were divided into two groups - Group A (Centchroman) and Group B (Evening primrose oil) each with 50 patients	Centchroman 30 mg orally twice weekly for the initial 3 months, followed by once weekly	Evening primrose oil 3 gm daily orally for of 3 months	1 year	Number of patients which showed complete response to treatment Centchroman group n=8 Evening primrose group n=1	Delay in menstruation Centchroman group n=3 Evening primrose group n=1 Scanty menses Centchroman group n=2 Evening primrose group n=0 Headache Centchroman group n=1 Evening primrose group n=1 Rash Centchroman group n=1 Evening primrose group n=0
Brahmachari et al. 2021 [26]	Prospective, single cohort, observational study; India	From June 2016 to July 2017	Patients aged ≤35 years with fibroadenoma	30 patients with multiple fibroadenoma (≥ 3), of size ranging from 0.5 to 3 cm.	Ormeloxifene 30 mg alternate day for 12 weeks	NA	6 months	Number of patients which showed complete regression of fibroadenoma n=9	Delayed menses in <10% of cycles
Dhar et al. 2007 [27]	Prospective single-arm observational study; India	August 2003 to September 2004	Patients aged up to 35 years with fibroadenoma <5 cm size	Total no. of patients enrolled, n=60 No. of patients in fibroadenoma group n=18	Centchroman 30 mg on alternate days for 3 months	NA	6 months	Number of patients which showed complete regression=7 (41%)	Allergic rash after second dose n=1 Delay in menstruation n=3 Complete amenorrhea n=2
Gupta et al. 2016 [28]	Prospective, interventional, uncontrolled study; India	From August 2011 to August 2014	Patients aged up to 35 years with history of mastalgia or fibroadenoma < 5 cm size	Total no. of patients enrolled (N=100). No. of women in fibroadenoma group n=29	Ormeloxifene 30 mg on alternate day for 3 months	NA	6 months	Number of patients which showed complete regression n=8	Out of 100 patients: Delayed menstruation n=14, Temporary amenorrhea n=8, Gastric upset n=8, Vague abdominal n=6, Headache n= 4 and Drug related rash n=2

Publication Bias

The funnel plot was used to assess publication bias in studies included in the meta-analysis. We did not retrieve enough studies to comment on publication bias.

Risk of Bias

**Figure 2:**
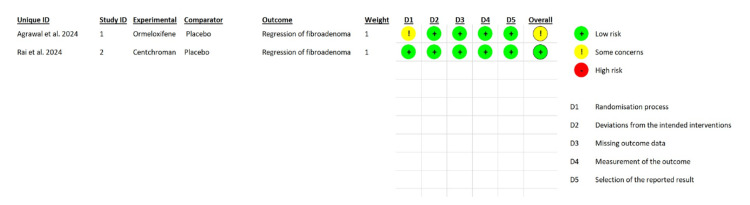
Traffic light plot: Author’s judgement of the risk of bias in randomized controlled trials (RCTs)

Efficacy Outcome

**Figure 3:**
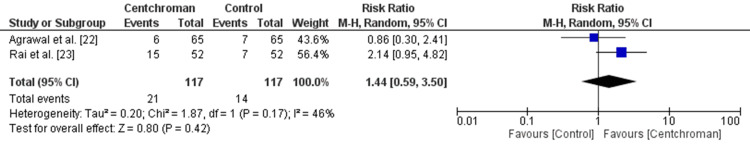
Forest plot for efficacy outcome

For meta-analysis of efficacy outcome, we performed pooling of two RCTs with pooled sample size of 234. In the RCTs conducted by Agrawal et al. [22], the efficacy outcome was regression of fibroadenoma and response to treatment was categorized into patients showing complete regression, partial regression and no regression. In the RCT conducted by Rai et al. [23], the efficacy outcome was reduction in volume of the largest fibroadenoma and patients showing at least 50% reduction in volume of the largest fibroadenoma were defined as responders by the authors. We harmonized the outcomes of two RCTs by defining patients who showed at least a 50% reduction in volume of fibroadenoma as responders. The pooled estimate (RR) of efficacy outcome measures of RCTs was 1.44 [95% CI 0.59 to 3.50; p=0.42]. Heterogeneity was found to be moderate with I2 value equal to 46% (Figure 3).

Safety Outcome

**Figure 4:**
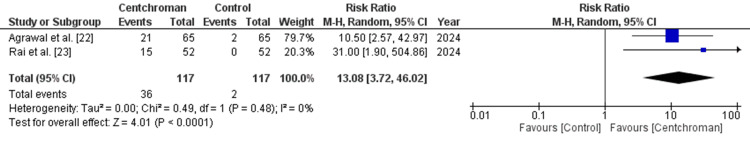
Forest plot for safety outcome

The pooled estimate (RR) of the safety outcome measure was 13.08 [95% CI 3.72 to 46.02; p=0.0001] (Figure 4). This signifies that patients who received centchroman developed a significantly higher number of adverse effects than those who received a placebo. Common adverse events reported in the included studies were menstrual irregularities such as scanty menstruation, delayed menstruation, or reversible amenorrhea; dizziness; headache; rashes; gastric upset and vague abdominal pain but none of the adverse events were serious.

Discussion

Several types of studies including RCTs have been conducted to evaluate efficacy and safety of centchroman in patients with breast fibroadenomas. The results of these studies were conflicting; some found centchroman efficacious while other did not. This systematic review and meta-analysis, to the best of our knowledge, is the first study attempting to resolve the conflict. Seven studies were included in the systematic review, out of which three were RCTs [22-24], three were prospective observational studies [25-27] and one was a single-arm interventional study [28]. We included only two [22,23] out of three retrieved RCTs in the meta-analysis because we could not harmonize the outcome in the third RCT [24] which was the number of fibroadenoma (rather than number of patients) showing regression. Four out of seven studies [22-25] included in this systematic review had a control arm whereas three [26-28] did not have any control. The control arm was either put on natural observation [24] or received a placebo [22,23] or an active drug, evening primrose oil [25]. The dose of centchroman used in all the studies was 30 mg. Out of seven studies, centchroman was given on alternate days for three months/12 weeks in five studies [22,23,26-28], once daily for 12 weeks in one study [24], and thrice weekly for the initial three months, followed by once weekly in one study [25]. The follow-up period in most studies was six months [22,26-28] or 24 weeks [24]; in one study it was 12 weeks [23] and in another study it was one year [25].

In both the RCTs included in the meta-analysis, the control arm received a placebo and the intervention arm received centchroman 30 mg orally on alternate days for three months. The follow-up period was six months in the RCT by Agrawal et al. [22] and 12 weeks in the RCT by Rai et al. [23]. Since in both studies, assessment of efficacy and safety outcomes was performed at the end of three months/12 weeks, the impact of variable follow-up period on efficacy and safety outcomes is unlikely. The pooled estimate (RR) of efficacy outcome measures of RCTs was 1.44 [95% CI 0.59 to 3.50; p=0.42] which suggests that centchroman does not cause a statistically significant difference in the regression of fibroadenomas between treatment and control arms. The finding of this meta-analysis is in the alignment of the double blinded-placebo controlled trial (The FIBROCENT study) conducted by Agrawal et al. [22], who found no significant difference in fibroadenoma regression rate between the ormeloxifene arm (9%) and the placebo arm (11%). However, this finding is against the finding of RCT conducted by Rai et al. [23] who in their study found that proportion of patients showing at least 50% reduction in volume of fibroadenoma was higher in centchroman arm (28.8%) than in placebo arm (13.5%) over a period of 12 weeks.

The pooled estimate (risk ratio, RR) of safety outcome measure was 13.08 [95% CI 3.72 to 46.02; p=0.0001]. This signifies that patients in the centchroman group developed a significantly higher number of adverse effects than those in the control group. The most common adverse effects were menstrual abnormalities such as missed menstruation and delayed menstruation. In the RCT conducted by Agrawal et al. [22], 32.2% patients in the ormeloxifene arm suffered from various adverse effects such as menstrual irregularity (15.4%), dizziness (12.3%), ovarian cyst (3%) and breakthrough bleeding (1.5%) as compared to 3% patients in placebo arm who suffered from menstrual irregularity whereas, in RCT conducted by Rai et al. [23], 28.8% patients in the centchroman arm had adverse effects in the form of scanty menstruation (17.3%) and delayed menstruation (11.5%) as compared to none in the placebo arm.

The mechanism of action of centchroman in regression of breast fibroadenoma is yet to be delineated. A probable mechanism can be antagonistic action on fibroadenoma which is corroborated by the presence of estrogen receptor on fibroadenoma tissue [29].

Strengths

This systematic review and meta-analysis, to the best of our knowledge is the first attempt to present a list of studies that evaluated centchroman for its efficacy and safety in treating breast fibroadenoma and to combine conflicting results of individual studies.

Limitations

This systematic review and meta-analysis have many limitations too. All the studies included in this systematic review and meta-analysis were conducted in India so their findings cannot be generalized to populations outside. Only two studies with smaller pool of patients in two arms were eligible for the meta-analysis, which may affect the generalizability of findings. As assessed by the RoB 2 tool, one of the two studies included in the meta-analysis had some concerns. The publication bias could not be assessed due to lack of availability of enough number of studies. The outcomes in the two RCTs included in the meta-analysis were slightly different which might have introduced heterogeneity in the data. Exclusion of studies in non-English languages and unpublished data may limit the comprehensiveness of evidence generated.

Recommendations

Owing to the limited number of studies involved in evidence generation, concern of risk of bias, and generalizability limited to Indian populations, the findings of this systematic review and meta-analysis should be used cautiously for clinical decisions and policy-making. In order to address above mentioned limitations, there is a need for a still greater number of high-quality RCTs with larger sample sizes to be conducted in different settings.

## Conclusions

Regression of fibroadenoma was not significantly higher in the centchroman group than the control group. Therefore, centchroman is not found to be more efficacious in causing regression of fibroadenoma than control. Furthermore, use of centchroman for breast fibroadenoma is associated with a significantly higher number of adverse effects than the control, although none of the adverse effects were serious. Menstrual irregularity was the most common adverse effect reported. Cenchroman, with this current level of evidence, cannot be an option for medical treatment for patients with breast fibroadenoma.

## Appendices

Search strategy

PubMed

Concept 1: Fibroadenoma (MeSH term)

Synonyms: Benign breast disease (older term)

Concept 2: Centchroman [MeSH term]

Synonym: Ormeloxifene

Search query: ((("fibroadenoma"[MeSH Terms] OR "fibroadenoma"[Title/Abstract] OR "benign breast disease"[Title/Abstract]) AND "centchroman"[MeSH Terms]) OR "centchroman"[Title/Abstract] OR "ormeloxifene"[Title/Abstract]) AND ((humans[Filter]) AND (1000/1/1:2024/12/31[pdat]) AND (english[Filter]))

Google scholar

Keywords: fibroadenoma, benign breast disease, centchroman, ormeloxifene

Advance search filters: 1. find articles with all of the words

                                      2.  anywhere in the article

Science Direct

Advanced search query: fibroadenoma OR "benign breast disease" AND centchroman OR ormeloxifene

Cochrane library

Advanced search query: fibroadenoma in Title Abstract Keyword OR "benign breast disease" in Title Abstract Keyword AND centchroman in Title Abstract Keyword OR ormeloxifene in Title Abstract Keyword

ClinicalTrial.gov

Expert search filters

Condition/disease: fibroadenoma

Other terms: benign breast disease

Intervention/treatment: centchroman OR ormeloxifene

Clinical Trial Registry-India (CTRI)

Advanced search option

Health condition/problem studied: fibroadenoma
